# Cyclodialysis cleft repair and cataract management by phacoemulsification combined with internal tamponade using modified capsular tension ring insertion

**DOI:** 10.1007/s00417-018-4149-8

**Published:** 2018-09-29

**Authors:** Jiahui Chen, Qinghe Jing, Wei Gao, Min Zhang, Yinghong Ji, Junyi Chen, Yongxiang Jiang, Yi Lu

**Affiliations:** 1 Department of Ophthalmology and Vision Science, Eye Ear Nose and Throat Hospital of Fudan University, 83 Fenyang Rd., Shanghai, 200031 China; 2 NHC Key Laboratory of Myopia (Fudan University), Laboratory of Myopia, Chinese Academy of Medical Sciences, Shanghai, China; 3 Key Laboratory of Visual Impairment and Restoration of Shanghai, Shanghai, China

**Keywords:** Cyclodialysis cleft, Cataract, Phacoemulsification, Modified capsular tension ring

## Abstract

**Purpose:**

To evaluate the surgical outcomes of cyclodialysis cleft repair and cataract management by phacoemulsification combined with internal tamponade using a modified capsular tension ring (MCTR) compared with direct cyclopexy.

**Methods:**

The preoperative and postoperative characteristics of patients with cyclodialysis clefts who underwent surgery via insertion of an MCTR into the ciliary sulcus (MCTR group; 16 patients, 16 eyes) or direct cyclopexy (DC group; 16 patients, 16 eyes) were recorded.

**Results:**

The cyclodialysis extended over 2.6 ± 1.9 clock hours in the MCTR group and 3.5 ± 1.8 clock hours in the DC group (*P* = 0.094). Postoperatively, the IOP was not significantly different between the MCTR and DC groups (12.9 ± 3.7 mmHg vs. 13.8 ± 6.2 mmHg, *P* = 0.985); the logarithm of the minimal angle of resolution BCVA was better (0.1 ± 0.2 vs. 1.0 ± 0.9, *P* < 0.001), and the anterior chamber depth was greater (3.87 ± 0.40 mm vs. 2.59 ± 0.58 mm, *P* < 0.001) in the MCTR group than in the DC group. Compared with the preoperative parameters, the postoperative BCVA, IOP, and anterior chamber depth values were significantly improved in the MCTR group (*P* < 0.05), whereas the BCVA showed no significant improvement postoperatively in the DC group (*P* = 0.174). Logistic regression revealed no significant risk factors for successful IOP control or BCVA improvement.

**Conclusion:**

Phacoemulsification combined with internal tamponade using MCTR insertion into the ciliary sulcus is a safe and minimally invasive method for effectively closing cyclodialysis clefts and managing cataract.

**Electronic supplementary material:**

The online version of this article (10.1007/s00417-018-4149-8) contains supplementary material, which is available to authorized users.

## Introduction

A cyclodialysis cleft forms by detachment of the longitudinal ciliary muscle fibers from the scleral spur, creating a secondary pathway for aqueous humor drainage into the suprachoroidal space, which may lead to persistent ocular hypotony [1, 2]. It can be caused by severe blunt trauma, or iatrogenically following procedures that involve surgical manipulation of the anterior segment [1, 3]. The ocular hypotony can lead to complications such as cataract progression, optic disc edema, shallow anterior chamber, refractive changes, choroidal effusion, and retinal fibrosis [4]. Accordingly, it is important to recognize cyclodialysis cleft and ensure timely intervention, using traditional treatment methods or novel noninvasive techniques [3, 5–11].

Various nonsurgical and surgical methods of closure have been proposed for the management of cyclodialysis cleft [1]. The nonsurgical options include medical treatment (steroids and atropine) [12], laser photocoagulation [13], transscleral diathermy [3, 14], and cryocoagulation [15, 16], whereas surgical procedures include direct cyclopexy [17], scleral buckling [18], internal tamponade [19], indirect cyclopexy [20], and novel therapeutic approaches [21, 22]. The principle underlying all of these strategies is to restore apposition of the detached ciliary body to the scleral spur using an internal or external procedure.

Recent studies that have reported cyclodialysis cleft treatment with internal tamponade by insertion of a capsular tension ring (CTR), modified CTR (MCTR), or intraocular lens (IOL) into the ciliary sulcus have shown satisfactory results [19, 23–26]. However, most published studies are limited to case reports and are too few in number to evaluate the surgical efficacy and safety. The purpose of this study is to report the surgical outcomes in a series of consecutive patients with cyclodialysis clefts and complicated cataract who underwent phacoemulsification and internal tamponade via MCTR insertion into the ciliary sulcus, compared with the outcomes in patients without cataract who underwent external direct cyclopexy, which is the gold standard for management of cyclodialysis cleft.

## Methods

### Patients and ethics

This prospective case-control clinical study recruited consecutive patients who attended the ophthalmology clinic and underwent surgery to repair cyclodialysis clefts at the Eye and ENT Hospital of Fudan University, Shanghai, China, from March 2016 to January 2018. A cyclodialysis cleft was defined as the detachment of the ciliary body root from the scleral spur as examined upon gonioscopy. The location and extent of the cyclodialysis cleft were confirmed by ultrasound biomicroscopy (UBM) (MD-300 L, MEDA, Tianjin, China). Patients of cyclodialysis cleft coexited with cataract were recruited in the MCTR group for undergoing surgery via phacoemulsification and internal tamponade with MCTR insertion into the ciliary sulcus, whereas patients of cyclodialysis cleft without cataract at the time of surgery were enrolled in the direct cyclopexy (DC) group for surgery of direct cyclopexy. The Human Research Ethics Committee of the Eye and ENT Hospital of Fudan University reviewed and approved this study. The research was conducted in accordance with the tenets of the Declaration of Helsinki. Written informed consent was obtained from all patients.

### Diagnostic techniques

Routine ophthalmic examinations including best-corrected visual acuity (BCVA), slit lamp examination, intraocular pressure (IOP), anterior chamber depth (ACD), fundus examination, and B-scan ultrasonography were performed for all patients. IOP was measured using non-contact tonometry (TX-20 Full Auto Tonometer, Canon Inc., Tokyo, Japan) three times, and the mean values were used in subsequent statistical analyses. Choroidal detachment and hypotony maculopathy were measured by B-scan ultrasonography and optical coherence tomography (OCT5000, Carl Zeiss Meditec, Inc., Dublin, CA, USA), respectively. To identify and confirm the extent and location of the cyclodialysis clefts, UBM was performed preoperatively and postoperatively.

### Surgical management

**Fig. 1:**
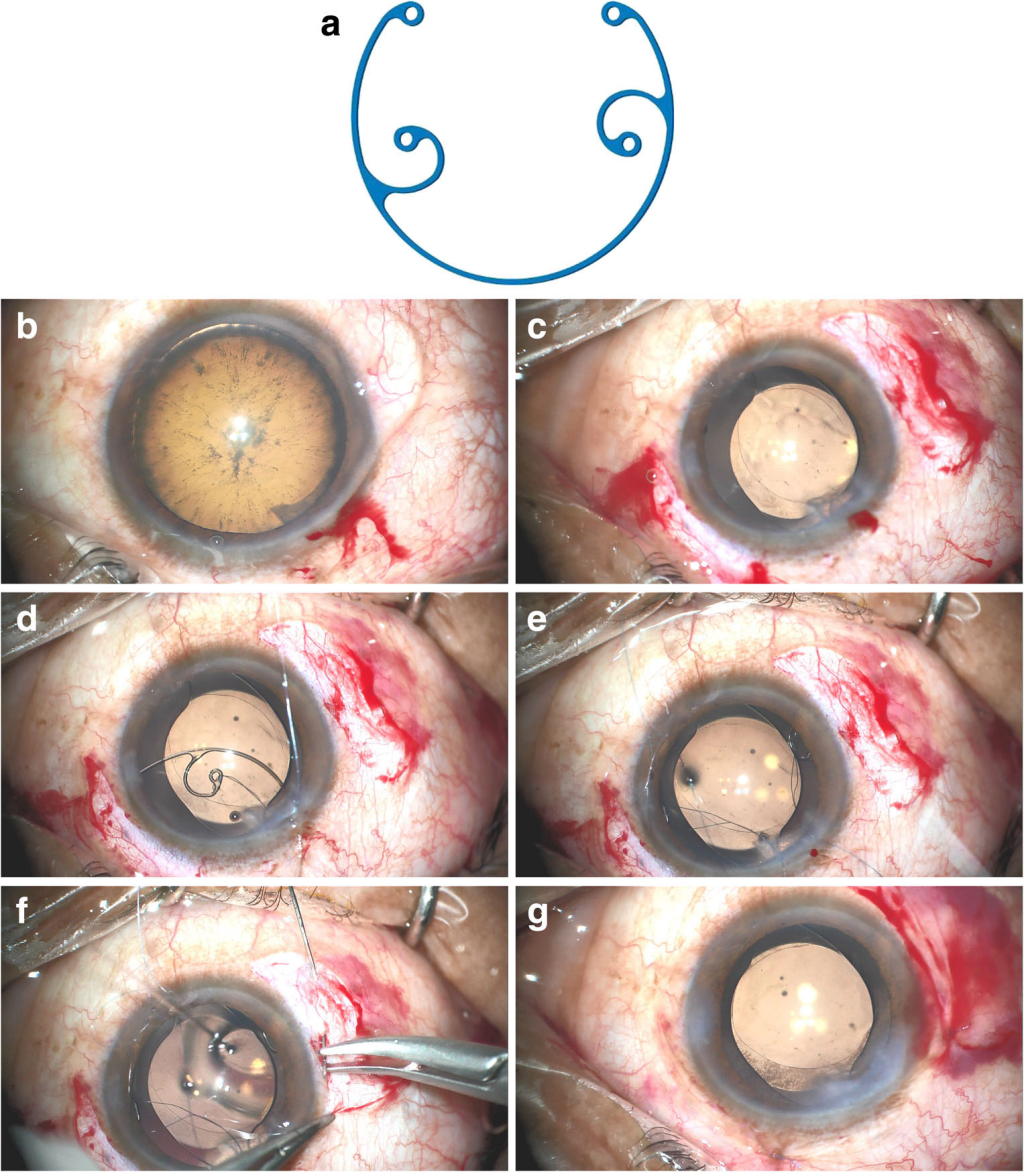
Intraoperative microscopic images of Cionni-modified capsular tension ring (MCTR) insertion in the ciliary sulcus. **a** A Morcher Type 2L MCTR, dimension: 11 mm (13 mm when stretched). **b** An illusion of lens subluxation and zonular dialysis caused by cyclodialysis cleft in the top left after filling the anterior chamber with viscoelastic material. **c** An oval capsulorhexis after phacoemulsification despite we made a round capsulorhexis. **d** A Morcher Type 2L MCTR with two eyelets preset with 10–0 polypropylene. **e** MCTR insertion into the ciliary sulcus. **f** MCTR sutured using 10–0 polypropylene to the sclera 1 mm posterior to the corneal limbus. **g** The capsulorhexis becoming round after cyclodialysis cleft repair by internal tamponade via MCTR insertion into the ciliary sulcus

All patients with persistent cyclodialysis clefts underwent surgery if nonsurgical treatment had been ineffective and if there were ocular complications. Patients in the MCTR group underwent phacoemulsification and MCTR insertion into the ciliary sulcus, as previously described by Jing et al. [26]. After making a clear corneal incision and a paracentesis, continuous curvilinear capsulorhexis and hydrodissection were performed (Fig. 1b, c). Following standard endocapsular phacoemulsification and cortical aspiration, a foldable IOL was implanted into the capsular bag with or without a CTR. An MCTR with two eyelets (Fig. 1a) preset with 10–0 polypropylene was then inserted into the ciliary sulcus and sutured to the sclera 1 mm posterior to the surgical limbus to mechanically reappose and reattach the detached ciliary body to the scleral spur (Fig. 1d–g). The position of the MCTR was adjusted to aim its maximum focal point at the site of the most severe cyclodialysis. In the control group (DC group), patients without dense cataracts at the time of surgery underwent DC for cyclodialysis cleft repair, as has been described previously [4]. After viscoelastic material was injected into the anterior chamber through a paracentesis to deepen the anterior chamber, the exact location and the extent of clefts were identified using gonioscopy. A limbal conjunctival peritomy and a thick lamellar limbus-based scleral flap were created. The remaining scleral floor was then incised posterior to the scleral spur, directly exposing the disinserted ciliary muscle. The needle was first passed through the anterior sclera just posterior to the scleral spur, then through the ciliary muscle, and finally through the posterior scleral. At the end, the scleral flap and the conjunctiva were closed.

### Outcome measurements

Preoperative data collection included age; sex; BCVA; IOP; ACD; the cause, extent, and location of the cleft; duration before surgery; and additional findings detected by B-scan ultrasonography, optical coherence tomography, and UBM.

Postoperative follow-up measurements, including BCVA, IOP, ACD, and duration of follow-up, were collected on postoperative days 1 and 3 and at 1, 3, and 6 months thereafter until the last clinical visit. Eyes with postoperative IOP spikes received antiglaucoma eye drops. The visual acuity measurements were converted to logarithm of the minimal angle of resolution (logMAR) notation. Successful IOP control was defined as an IOP of 10–21 mmHg.

### Statistical analysis

Statistical analyses were performed using SPSS version 23.0 (IBM Corp., Armonk, NY, USA). Descriptive statistics included the mean ± standard deviation and range where appropriate. A normal data distribution was confirmed using the Shapiro–Wilk test. Student’s *t* test, chi-square test, and Wilcoxon rank-sum test (Mann–Whitney *U* test) were applied as appropriate for comparisons between the two groups. The paired Student’s *t* test was used to compare the preoperative with the postoperative measurements within the same group. Logistic regression was applied to determine the associations of IOP recovery and BCVA improvement with age, sex, duration before surgery, preoperative parameters, and extent of the clefts. The results of two-sided tests were considered significant at *P* < 0.05.

## Results

The MCTR group included 16 patients (16 eyes) with cataracts (mean age, 51.9 ± 8.5 years (range, 31–65 years)). The DC group included 16 patients (16 eyes) without cataracts at the time of surgery (mean age, 47.8 ± 11.9 years (range, 30–74 years) and a male predominance, 68.8%) (Table 1). The preoperative data are listed in Table 1. In both groups, the clefts were caused by trauma in 14 eyes and by previous ocular surgery in the other 2 eyes. In the MCTR group, surgical repair was performed at 7.6 months after the trauma or iatrogenic injury (range, 15 days to 24 months); in the DC group, surgical repair was performed at 4.3 months (range, 21 days to 12 months) (*P* = 0.175). The numbers of clefts and their extents were not significantly different between the two groups (*P* = 1.000 and *P* = 0.094, respectively). Other than cyclodialysis cleft, concomitant complications included lens subluxation (< 120°; *n* = 3 eyes), optic disc edema (*n* = 13 eyes), cataract (*n* = 14 eyes), and hypotony maculopathy (*n* = 7 eyes) in the MCTR group and included pseudophakic eyes (*n* = 2 eyes), recession of the anterior chamber angle (*n* = 3 eyes), optic disc edema (*n* = 13 eyes), hypotony maculopathy (*n* = 11 eyes), iridodialysis (*n* = 1 eye), and retinal detachment (*n* = 1 eye) in the DC group.

**Table 1:** Preoperative clinical characteristics of patients with cyclodialysis clefts

	Group		*P* value
	MCTR	DC	
No. eyes	16	16	
Age (year)	51.9 ± 8.5	47.8 ± 11.9	0.260
Gender (male/female)	11/5	11/5	1.000
Right/left	12/4	7/9	0.072
Cause (trauma/surgery)	14/2	14/2	1.000
Duration (months)	7.6 ± 6.7	4.3 ± 4.0	0.175
No. cleft	1.1 ± 0.3	1.1 ± 0.3	1.000
Extension (clock hours)	2.6 ± 1.9	3.5 ± 1.8	0.094
BCVA (logMAR)	1.1 ± 0.7	1.3 ± 0.8	0.559
IOP (mmHg)	9.9 ± 3.1	8.1 ± 2.5	0.039^a^
ACD (mm)	1.88 ± 0.49	2.20 ± 0.61	0.112

*MCTR*, modified capsular tension ring; *DC*, direct cyclopexy; *BCVA*, best-corrected visual acuity; *logMAR*, logarithm of the minimal angle of resolution; *IOP*, intraocular pressure; *ACD*, anterior chamber depth

^a^Mann–Whitney *U* test

**Fig. 2:**
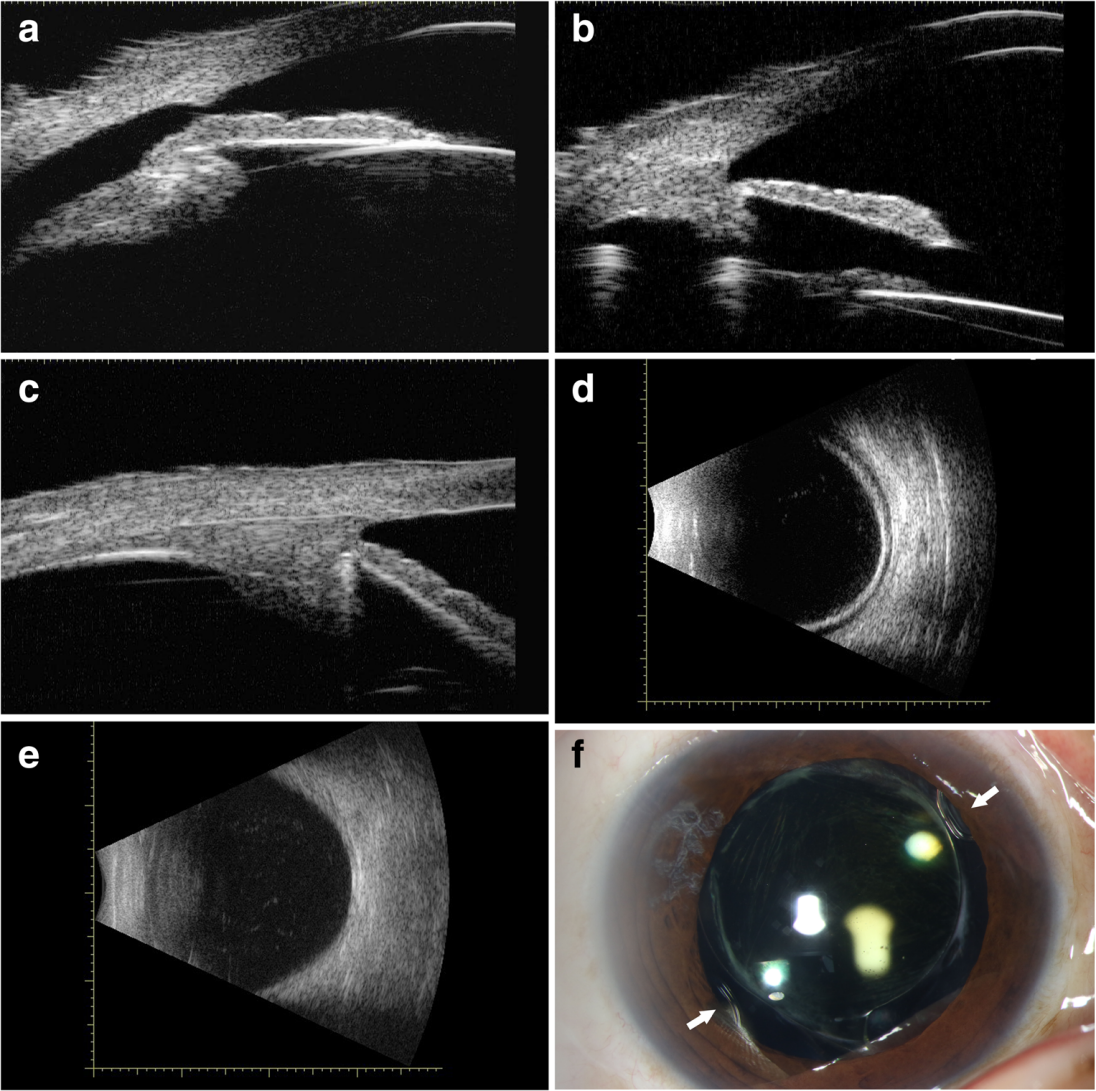
Representative ultrasound biomicroscopic (UBM), B-scan ultrasonographic, and photographic images in the Cionni-modified capsular tension ring (MCTR) group. **a** Preoperative UBM image of cyclodialysis cleft. **b** Postoperative UBM image showing closure of the cyclodialysis cleft and “dual indentation sign” on the ciliary body by the MCTR in the ciliary sulcus and capsular tension ring in the capsular bag. **c** Postoperative UBM image showing single indentation sign by the MCTR in the ciliary sulcus. **d** B-scan ultrasonographic image showing choroidal detachment before cleft closure. **e** B-scan ultrasonographic image showing the disappearance of choroidal detachment after cleft closure. **f** Photograph shows the MCTR positioned stably in the ciliary sulcus; the white arrows indicate the two eyelets

The postoperative mean follow-up period was 6.6 ± 5.7 months in the MCTR group and 8.2 ± 6.7 months in the DC group, with a range of 1–20 months for both groups (*P* = 0.515; Table 2). At the end of follow-up, cleft closure was confirmed by UBM, and the disappearance of choroidal detachment was confirmed by B-scan ultrasonography in 10 eyes (62.5%) of the MCTR group and in 10 eyes (62.5%) of the DC group (Fig. 2). In the MCTR group, postoperative clinical images showed the MCTR positioned stably in the ciliary sulcus with 2 eyelets sutured to the sclera (Fig. 2). Postoperatively, the IOP returned to normal in 12 eyes (75.0%) of the MCTR group and in 10 eyes (62.5%) of the DC group, at different times after surgery. An IOP spike occurred in six eyes (37.5%) of the MCTR group and in seven eyes (43.8%) of the DC group within a few days after surgery, and this temporary elevation of the IOP could be controlled in all eyes by topical application of antiglaucomatous medication. Only one eye of the MCTR group had an IOP spike greater than 50 mmHg, and drainage of aqueous humor was performed from a lateral incision on postoperative day 2. The CTR was inserted into the capsule to manage lens subluxation in three patients (three eyes) in the MCTR group (Fig. 2).

**Table 2:** Postoperative clinical characteristics of patients with cyclodialysis clefts

	Group		*P* value
	MCTR	DC	
Follow-up (month)	6.6 ± 5.7	8.2 ± 6.7	0.515
BCVA (logMAR)	0.1 ± 0.2	1.0 ± 0.9	< 0.001^a^
IOP (mmHg)	12.9 ± 3.7	13.8 ± 6.2	0.985
ACD (mm)	3.87 ± 0.40	2.59 ± 0.58	< 0.001^b^

*MCTR*, modified capsular tension ring; *DC*, direct cyclopexy; *BCVA*, best-corrected visual acuity; *logMAR*, logarithm of the minimal angle of resolution; *IOP*, intraocular pressure; *ACD*, anterior chamber depth

^a^Mann–Whitney *U* test

^b^Student’s *t* test

The mean preoperative IOP was 9.9 ± 3.1 mmHg (range, 6.0–15.3 mmHg) in the MCTR group and 8.1 ± 2.5 mmHg (range, 5.0–14.5 mmHg) in the DC group (*P* = 0.039; Table 1), whereas the mean postoperative IOP was not significantly different between the two groups (12.9 ± 3.7 mmHg vs. 13.8 ± 6.2 mmHg, *P* = 0.985; Table 2). Compared with the preoperative measurements, the IOP was increased significantly in both groups postoperatively (*P* = 0.017 for the MCTR group and *P* = 0.006 for the DC group; Table 3). At the final visit, the BCVA was 0.1 ± 0.2 logMAR, which had improved significantly, in the MCTR group (*P* < 0.001; 95% confidence interval, 0.59–1.12). However, the mean BCVA remained unchanged in the DC group postoperatively compared with preoperatively (1.0 ± 0.9 logMAR vs. 1.3 ± 0.8 logMAR, *P* = 0.174). None of the eyes had no light perception. The UBM results confirmed cleft closure and revealed a deeper ACD postoperatively in the MCTR group (3.87 ± 0.40 mm, *P* < 0.001) than in the DC group (2.59 ± 0.58 mm, *P* = 0.006) (Table 3).

**Table 3:** Preoperative and postoperative values in the modified capsular tension ring and direct cyclopexy groups

	Group					
	MCTR		*P* value	DC		*P* value
	Pre	Post		Pre	Post	
BCVA (logMAR)	1.1 ± 0.7	0.1 ± 0.2	< 0.001^a^	1.3 ± 0.8	1.0 ± 0.9	0.174
IOP (mmHg)	9.9 ± 3.1	12.9 ± 3.7	0.017^a^	8.1 ± 2.5	13.8 ± 6.2	0.006^a^
ACD (mm)	1.88 ± 0.49	3.87 ± 0.40	< 0.001^a^	2.20 ± 0.61	2.59 ± 0.58	0.006^a^

*MCTR*, modified capsular tension ring; *DC*, direct cyclopexy; *Pre*, preoperative; *Post*, postoperative; *BCVA*, best-corrected visual acuity; *logMAR*, logarithm of the minimal angle of resolution; *IOP*, intraocular pressure; *ACD*, anterior chamber depth

^a^Paired Student’s *t* test

Logistic regression analysis revealed no statistically significant associations of age, sex, preoperative IOP, preoperative BCVA, preoperative ACD, number and extent of the clefts, IOP spike, or hypotony maculopathy with an increased IOP or improved BCVA after surgical intervention. In the MCTR group, potential risks of ciliary body damage, erosion, hemorrhage, and pain from the compressive effect of the MCTR were not observed; however, posterior capsulotomy using a Nd:YAG laser was performed for posterior capsule opacification in two eyes. Five patients (five eyes) in the DC group underwent a secondary intervention during the postsurgical follow-up period for cataract progression.

## Discussion

Cyclodialysis clefts and spontaneous reattachment are rare. Therefore, lots of methods have been proposed for the reattachment of the longitudinal ciliary muscle fibers from the scleral spur. The first-line therapy for small clefts is atropine and steroids, and previous studies have reported a good prognosis in small-sized cyclodialysis clefts at under 1.5 clock hours following argon laser photocoagulation treatment [4, 13, 27]. Clefts that show a poor response to nonsurgical treatment should be identified accurately and treated appropriately with feasible surgical strategies.

Surgical options for cyclodialysis cleft repair include direct cyclopexy; scleral buckling; pneumocyclopexy; vitrectomy; sulcus CTR, MCTR, or IOL insertion; and gas tamponade [13, 17–19]. Direct cyclopexy, the gold standard for treatment of large clefts, is probably the most effective approach to close a cyclodialysis cleft, but it has some limitations, such as severe intraoperative hypotony, potential complications, the need for careful surgical procedures, damage to vascular structures, disrupted filtering bleb function after trabeculectomy, and a long recovery period after surgery [17, 24]. After direct cyclopexy, most patients require a secondary intervention related to the presence of complicated cataract, lens subluxation, vitreous prolapse, or other small clefts.

Since the surgical technique for cataract extraction and repairing a 360° cyclodialysis cleft using phacoemulsification and insertion of a CTR in the ciliary sulcus was first proposed [24], many surgeons have attempted applying an IOL or MCTR to mechanically reappose the detached ciliary body to the scleral spur [19, 25, 26, 28]. In the present cases requiring cataract surgery, lens subluxation management and cleft repair, multiple procedures were performed simultaneously including phacoemulsification, implantation of IOLs, and insertion of an MCTR in the ciliary sulcus. In terms of the postoperative outcomes, the BCVA improved and ACD increased significantly in the MCTR group compared with the DC group because of the contributions of trauma-related cataract, lens subluxation, and hypotonic maculopathy to the postoperative visual performance.

Compared with direct cyclopexy, the MCTR insertion surgical technique described here is associated with simpler manipulation, a quicker recovery, and less expense because of the small incision, routine technique, and minimal suture, which is more familiar for cataract surgeons [19, 26]. However, because of the lack of accommodation after IOL implantation, we recommend performing direct cyclopexy for patients with clear lenses and conducting long-term follow-up to monitor the development of cataracts in the future.

Although surgical management had a higher success rate in the MCTR group, four patients whose BCVA had improved still had a low IOP (7.8–10.0 mmHg). Of these four eyes, three underwent surgery within 3 months. UBM showed closure of the most severe clefts, but small clefts remained, and required a long time to recover. Therefore, laser photocoagulation would be considered in these patients, along with long-term follow-up. In addition, UBM revealed clefts in two patients in the MCTR group whose IOP was normal. We speculated that the production and outflow rate of aqueous humor into the trabecular meshwork and suprachoroidal space had reached equilibrium in these two patients. In the DC group, four eyes had a low IOP (6.7–10.0 mmHg), three of which still had clefts confirmed by UBM after postoperative 3 months. We plan to perform MCTR insertion and phacoemulsification in these three patients with cataract progression. In addition, two patients whose IOP was high (29.9 mmHg and 24.6 mmHg) were treated with antiglaucomatous medication and needed a continuous follow-up.

To identify statistically significant risk factors, we performed logistic regression to analyze the associations between successful IOP control or BCVA improvement and preoperative and postoperative parameters but found no statistically significant potential risk factors. However, in the DC group, BCVA did not improve significantly after surgical intervention. Visual outcome depended on the traumatic complexity and severity of trauma-related complications. We acknowledge that hypotonic maculopathy and cataract progression may contribute significantly to visual acuity improvement, as the extent of the clefts was larger in the DC group, and more of those eyes had hypotony maculopathy.

According to the outflow rate of aqueous humor into the suprachoroidal space, hypotony (IOP ≤ 5 mmHg) did not always occur in patients with cyclodialysis clefts, regardless of the size of the cleft. Although their IOP was greater than 5 mmHg, our patients underwent surgery to repair the clefts and other ocular complications after ineffective nonsurgical treatments. We recommend persistent hypotony after trabeculectomy in which the incision is too back to damage longitudinal ciliary muscle fibers from the scleral spur resulting in the cyclodialysis cleft to be an indication for MCTR insertion into the ciliary sulcus. A second indication is a cyclodialysis cleft with complicated cataract and/or lens subluxation. Finally, this surgical approach may be an option in cases of failed cleft closure by direct cyclopexy due to a large extent of cleft involvement and cataract progression.

Other surgical methods of internal tamponade including IOL insertion into the ciliary sulcus and gas tamponade had shown satisfactory results reported by many studies. When placing an IOL in the ciliary sulcus, the stiff haptics of the IOL can exert some force against the ciliary body to the sclera spur, leading to closure of the cyclodialysis cleft in that area [25, 28]. However, stiff IOL haptics may cause long-term damage to the ciliary body such as erosion, hemorrhage, pain, and inflammation. In addition, the haptics may not be enough and exact to provide support for a larger cleft. As for gas tamponade, it may be more suitable for cyclodialysis cleft patients coexisted with vitreoretinal surgery. Moreover, the rate of gas absorption and the reattachment of clefts may not be synchronized. To avoid more serious complications and promote more effective reattachment, CTR or MCTR insertion in the ciliary sulcus is advisable [29]. For optimal compliance with the diameter of the ciliary sulcus, we propose that a 13-mm-diameter MCTR with two-eyelet transscleral suture fixation is appropriate, as the anatomical diameter of the ciliary sulcus is approximately 12.5 mm in nearly emmetropic adult eyes [23, 29, 30].

Closure of a cyclodialysis cleft, recovery of aqueous humor production by the ciliary body, and partial recovery of trabecular meshwork function can result in an IOP spike [28]. The early IOP spike that occurred in six eyes of the MCTR group and seven eyes of the DC group returned to normal levels with functional recovery of the trabecular meshwork and with medication. As the contact between the eyelets of the MCTR and the iridial posterior surface was considered to be the cause of uveitis and the IOP spike, it was postulated that a CTR without eyelets is a better choice than an MCTR [24]; however, we believe that an MCTR has more advantages. First, MCTRs with eyelets provide better stability. Second, the position and tightness of the MCTR can be adjusted to aim the maximum focal point to the site of most severe cyclodialysis for the greatest tamponade effect. Finally, localized inflammatory reactions caused by direct suture of the MCTR eyelets can increase adhesion and promote cleft closure, whereas the CTR may not adhere sufficiently to the ciliary body to exert an internal tamponade effect.

There are two limitations in our study. First, the study participants were patients with cyclodialysis clefts who underwent MCTR insertion or direct cyclopexy without comparison with patients exhibiting other ocular pathologies, which may have introduced bias. Second, this was a short-term study that did not investigate the long-term curative effects of the two different surgical methods.

Although the surgical approach of inserting a CTR, MCTR, or IOL into the ciliary sulcus to repair traumatic or iatrogenic cyclodialysis clefts has been reported previously, the current study of a relatively large number of consecutive patients with cyclodialysis clefts reported a technique that showed a good prognosis, similar to that achieved by conventional direct cyclopexy. In summary, in the setting of cyclodialysis clefts, coexisting cataracts, and/or lens subluxation, phacoemulsification accompanied by IOL implantation and internal tamponade using MCTR insertion into the ciliary sulcus is a safe and minimally invasive method to effectively close cyclodialysis clefts and manage cataracts and/or lens subluxation.

## Electronic supplementary material

ESM 1 Cyclodialysis Cleft Repair and Cataract Management by Phacoemulsification Combined with Internal Tamponade Using Modified Capsular Tension Ring Insertion into the ciliary sulcus. (MP4 113694 kb)
